# Syphilis of the Eye and Ear

**Published:** 1886-07

**Authors:** R. O. Cotter

**Affiliations:** Macon, Ga.


### SYPHILIS OF THE EYE AND EAR.

READ AT A MEETING OF THE MACON MEDICAL SOCIETY, BY R. O. COTTER, M. D., MACON, GA.

Syphilitic lesions of the external ear are so rare as to hardly warrant a notice in as short a paper as this must necessarily be. They are usually limited to ulcers or gummata of the auricle, and are, when found at all, usually met with along with other well known symptoms in the secondary stage, and of course are to be treated upon general principles. Syphilis of the middle ear, viz., the cavity of the tympanum, the ossicula audita, the mastoid cells, and the eustachian tube, is quite the commonest form in which the disease affects the ear. Either, 1st, as a result of syphilitic nasal catarrh, having gradually extended up the eustachian tubes, or 2nd, as frequently occurs, a gradual proliferation of tissue of the living membrane of the tympanum, causing thickening of the membrana tympani with ankylosis of the ossicula and their adhesion to the walls of the tympanum. In either case we call it syphilitic aural catarrh. In the last mentioned form the symptoms frequently begin as in any case of acute aural catarrh from a cold perhaps. In all probability the patient would have had the attack of catarrh without having syphilis, but the syphilitic dyscrosia of course tends to hasten the trouble, and thus constitutes a syphilitic catarrh of the middle ear. It is important that we should differentiate between this disease of the sound-conducting apparatus and that of the sound-receiving apparatus, *i. e.* the auditory nerve in the labyrinth. We know that the tuning-fork is the proper means of making the test. I have generally found syphilitic aural catarrh to occur as a tertiary symptom or a late secondary symptom—say in from two years and upwards from the appearance of the chancre. I can best illustrate the symptoms by giving the following history of a case, which is the only case of syphilitic middle ear catarrh to more than a score of non-specific cases that I have under treatment at present: A man thirty years old came to me late in December for treatment. He had nasal catarrh with quite a profuse discharge. Left ear normal. Right ear was able to hear only very loud and close conversation, no pain, but a most annoying tinnitus aurium in right ear, which he compared to the sound of pouring water. Right ear "felt stopped up;" examination of the ear showed that the drum membrane was sunken in, and of the leaden hue usual in aural catarrh. The tuning-fork, when placed on top of his head, was heard best in the bad ear, though when it was placed before the ears it was heard best in the good ear. This of course confirmed the diagnosis of disease of the middle ear, and while he at first denied it, I soon got a satisfactory syphilitic history. Inflammation of the middle ear with air, and chloroform vapor with the eustachian catheter, soon reduced the drum membrane (for the time at least) to its proper position, which instantly greatly mitigated all his symptcms.

This treatment has been kept up since at intervals of two or three days. I have had him on pretty full doses of iodide of potash with inunctions of mercury, and have had him use Labarraque's solution and warm salt water thrown through the nostrils with a Warner's post-nasal douche'. All his symptoms are rapidly improving.

This being a recent case, I think it quite likely that his hearing will be almost entirely restored or greatly improved. Though, if such cases are of over several months' standing, the prognosis is most unfavorable.

Perhaps congenital syphilis of the ear is most generally manifested in the middle ear. Along with other well known symptoms of inherited syphilis, such children usually become affected with nasal catarrh, and the disease extends up the eustachian tubes. In such cases they should be treated by proper anti-syphilitic measures, and the ear trouble can be treated by Politzer's method, which is simple and well known.

Coming now to the consideration of syphilis of the internal ear, which, while it frequently is a secondary result of syphilitic middle ear catarrh, is also found—confirmed by pathological investigation—aside from the latter. Perhaps the cochlea is generally the exact seat of disease. It probably occurs oftener within the first year or two of syphilis, though it may come on later as a syphilitic periostitis.

In these cases the patient will usually be able to definitely locate the time of the beginning of the dullness of hearing, tinnitus, etc. If the hearing is not entirely gone—that is for the watch or fork, at least—the patient will hear the tuning-fork better when placed in front of the ear than when placed on the top of his head, or mastoid process, which goes to show that the sound-receiving apparatus, the auditory nerve, is affected, because in this case the external auditory canal and tympanum are not diseased, and are consequently the best and natural channel of sound-conduction.

If these cases are diagnosed early and followed up with active and proper anti-syphilitic treatment, the prognosis is rather favorable. Inflation of the tympanum here with the eustachian catheter, or by any other means, is calculated to do harm rather than good.

Syphilis of the eye manifests itself most frequently in the form of iritis, and more than one-half the cases of iritis not traumatic are syphilitic. Besides the well known usual symptoms of iritis, there will be very frequently gummy tumors on the iris and generally toward the pupillary edge. The aqueous humor is turbid, and the posterior layer of the cornea is hazy with lymph. The pupil is likely to become occluded with lymph. The iris becomes adhered to the lens and is likely to leave a firm covering of pigment over the capsule which can never be removed, and frequently leads to irremediable blindness. The retina becomes more or less implicated, as it is now known to be in any case of iritis, and is apt to leave the vision more or less clouded. If the iritis is not checked, choroiditis or irido-cyclo-choroiditis is apt to follow, with softening and atrophy of the ball. It seems that no practitioner now-a-days ought to overlook the fact that syphilis is so frequently the cause of iritis, yet I am constantly meeting with such cases, and by far the largest majority of cases of syphilitic iritis which I treat are those cases occurring in from six months to two years after the patient had chancre, and they nearly always tell me that they took medicine for one to three months by their physicians' directions. Now why is this? Is it because their physicians err in not having them follow up the treatment fully, or is it because they haven't the courage to refuse to promise a patient a cure in one to three months? I am constantly meeting with such cases, and it is thoroughly apparent that too many practitioners are not searching enough in their inquiries as to syphilitic symptoms and history.

Perhaps the next most frequent syphilitic affection of the eye is diffuse corneitis from inherited syphilis. It is very common among negroes, and generally manifests itself along with other symptoms of syphilis, and very frequently in such children as I referred to in speaking of syphilitic aural catarrh, though it may occur at any time up to the 15th year. It may be also from acquired syphilis. The cornea is hazy like a mirror upon which the breath has been blown. It is not usually an inflammatory or painful disease, though there is occasionally severe photophobia. One peculiarity is that in the congenital form the cornea is not at all likely to ulcerate unless improperly treated—that is, by strong astringents or caustics.

Now, in syphilitic phlyctenular corneitis, which is also quite common, there is a decided tendency to ulceration, though the treatment in these two diseases is very similar. If it is a case of syphilitic corneitis in a child unweaned, we should direct it to be nourished by a healthy woman, if possible, and use oleate of mercury, rubbed in a flannel bandage, kept applied around the child's belly. As the child grows older give it syr. iod. iron in from 3 to 20-drop doses.

Good nourishing diet and abstinence from trashy food or sweetmeats is of the utmost importance. Cod-liver oil is of very great value here. Use a ¼ to 2 gr. sol. of atropia daily in the eyes. Yellow oxide of mercury ointment, ½ to 2 gr. to the drachm, applied to the edges of the eyelids, with the application three or four times daily of hot water on the outside of the closed lids, is useful to promote absorption of the lymph in the cornea. With this treatment kept up faithfully for three to six or eight months, the prognosis is quite favorable.

Syphilitic choroiditis is rather common. The ophthalmoscope generally reveals it in the form of patches of atrophied choroid surrounded by black fringes of pigment. Even with active treatment the prognosis is more or less unfavorable according to the extent of the patches.

Syphilis very frequently attacks the nerves of the eyes, and especially the third pair, causing ptosis, and especially causes outward movement of the ball, and also causes inability to move the ball inward past the median line. It also frequently is the cause in this way of double vision. The prognosis ought to be favorable if the case is recent. Of course large doses of iod. pot., followed by strychnine, is the thing here. Atrophy of the optic nerve, caused by syphilitic deposits on the optic tracts within the cranium, is also common.

Strictures of the nasal duct, with and without lachrymal fistulæ, caused from syphilis, is quite common, as was illustrated by the typical case I reported at our last meeting.

With proper operative procedures, followed by active anti-syphilitic treatment, these are easily curable.

My experience is that if stricture of the nasal duct in a child does not yield to astringents without an operation, it is usually caused by congenital syphilis. Now, permit me to anticipate the discussion of our subject for the evening, "General Syphilis," by giving a short opinion as to the diagnosis and treatment of syphilis. Four or five years ago I had watched syphilis so closely that I thought I could distinguish between a syphilitic chancre and a chancroid. I now believe that they are so frequently mixed sores, or at least I now feel that experience has taught me that even with a satisfactory history from the patient, we are about as unable to predict what will follow any usual venereal sore as to guess what will be the verdict of a petit jury.

What cases I have treated I have watched very carefully. The following case will illustrate my usual plan when I was in general practice: A gentleman, aged 29, consulted me, December 25, 1882, with a typical-looking single chancre, which had appeared on the frenum, 22 days after exposure. Having his complete confidence, I inoculated him on the breast with matter from the chancre. It never took. I then destroyed the chancre with nitric acid, explaining to him thoroughly that I did not do so to cure his disease, but as there was a possibility of its being a mixed sore, and also to prevent his infecting some woman, and also to have him rid of the sore, I destroyed it. I made him wait for secondary symptoms, which came on in January in the form of enlarged inguinal glands, sore throat and papular eruptions over the body. I then began him upon mercurial inunctions and one-sixteenth grain, gradually increased to one-twelfth grain of corros. chlo. mercury, with 20 to 30 grains iod. pot. and soon settled down to one-sixteenth grain of the corros. chlo. with ten to fifteen grains iod. pot., and kept him steadily on it up to March, 1885. He fattened on the treatment, and has not had a syphilitic symptom since July, 1883. Whether he will have or not I tell him I don't know, but I don't believe he will.

I believe we should treat syphilis for two and a half to three years.

I have recently had the following case of syphilitic affection of the internal ear or acoustic nerve under treatment: A young man, aged 22, consulted me on January 16 for treatment of syphilitic iritis in both eyes. He had had chancre one year previously, but had taken no medicine of consequence, except some patent blood remedy. He was put upon pretty full doses of iod. potash, and mercurial inunctions were employed, together with the usual local treatment. By the 10th of March both eyes were well.

On March 21st, he awoke one morning with paralysis of the left facial nerve. The muscles of the cheek of that side swagged, and he could not whistle. His intellectual faculties were not at all impaired, though he staggered as he walked. He was also troubled with a most annoying buzzing noise, only in the left ear, which he compared to the hissing of steam. When the right ear was firmly closed he was perfectly deaf to conversation; could not hear the watch tick at all. He could not hear the tuning-fork by bone-conduction—that is, when placed upon his head. There was no abnormal appearance whatever of the external canal or drum membrane, nor did the two or three days' inflation of the eustachian tube of that side (which I employed merely to confirm my diagnosis) make any impression whatever upon his symptoms. He had a slight neuralgic pain of the fifth nerve of the temporal region of the affected side. It was plainly and unmistakably syphilitic affection of the labyrinth.

The same treatment as for the eye trouble (which, by the way, he had left off in spite of my advice) was kept up. The iod. potash was rapidly increased 60 to 80 grs. to the dose. The symptoms showed very little improvement until about the 12th of April, when he could, with the right ear stopped firmly, hear conversation tolerably well out of the affected ear. He could also hear the watch at three inches. The paralysis of the seventh nerve had very nearly disappeared and the pain all gone. I feel quite hopeful of this patient's ultimate recovery.

