# A Correction

**Published:** 1886-08

**Authors:** 


A CORRECTION.—In THE JOURNAL for July, on page 290, first line, living should be *lining;* same page, fifth and sixth lines from bottom, inflammation should be *inflation.*

